# Near-infrared upconversion multimodal nanoparticles for targeted radionuclide therapy of breast cancer lymphatic metastases

**DOI:** 10.3389/fimmu.2022.1063678

**Published:** 2022-12-01

**Authors:** Chuan Zhang, Yujuan Zhang, Maolin Liang, Xiumin Shi, Yan Jun, Longfei Fan, Kai Yang, Feng Wang, Wei Li, Ran Zhu

**Affiliations:** ^1^ State Key Laboratory of Radiation Medicine and Protection, School of Radiation Medicine and Protection, Collaborative Innovation Center of Radiation Medicine of Jiangsu Higher Education Institutions, Soochow University, Suzhou, Jiangsu, China; ^2^ Department of Nuclear Medicine, Nanjing First Hospital, Nanjing Medical University, Nanjing, China; ^3^ Department of Pathology, Experimental Center of Suzhou Medical College of Soochow University, Suzhou, Jiangsu, China; ^4^ The Affiliated Suzhou Hospital of Nanjing Medical University, Suzhou Municipal Hospital, Gusu School, Nanjing Medical University, Suzhou, Jiangsu, China; ^5^ Department of General Surgery, The Second Affiliated Hospital of Soochow University, Suzhou, Jiangsu, China

**Keywords:** breast cancer, lymph node metastasis, multimode imaging, radiotherapy, theranostic nanoplatform

## Abstract

The theranostics of lymph node metastasis has always been one of the major obstacles to defeating breast cancer and an important decisive factor in the prognosis of patients. Herein, we design NaGdF_4_:Yb,Tm@NaLuF_4_ upconversion nanoparticles with PEG and anti-HER2 monoclonal antibody (trastuzumab, Herceptin) (NP-mAb), the delivery of NP-mAb through the lymphatic system allows for effective targeting and accumulation in lymphatic metastasis. Combination of radionuclides ^68^Ga and ^177^Lu could be chelated by the bisphosphate groups of NP-mAb. The obtained nanoprobe (NP-mAb) and nanonuclear drug (^68^Ga-NP-mAb or ^177^Lu-NP-mAb) exhibited excellent stability and show high accumulation and prolong retention in the lymph node metastasis after intratumoral injection into the foot pad by near-infrared fluorescence (NIRF), single-photon emission computed tomography (SPECT) and positron emission tomography (PET) imaging. Utilizing the β-rays released by ^177^Lu, ^177^Lu-NP-mAb could not only decrease the incidence of lymph node metastasis, but also significantly decrease the volumes of lymph node metastasis. Additionally, ^177^Lu-NP-mAb induce no obvious toxicity to treated mice through blood routine, liver and kidney function assay. Therefore, nanoprobe and nanonuclear drug we designed could be acted as excellent theranostics agents for lymph node metastasis, providing potential alternatives diagnose and treatment option for lymph node metastasis.

## Introduction

Tumor metastasis is an extremely severe step in the progression of tumors and approximately 90% of deaths in cancer patients are due to metastasis rather than the primary tumor (1). The lymphatic system is the preferred route for metastasis of most solid tumors *in vivo*, and draining lymph nodes are usually the earliest sites and the first station of metastasis (2, 3). Lymph node metastasis often indicates a poor prognosis, and is an important indicator of cancer progression (4, 5). The dissection and local radiotherapy of sentinel lymph nodes can significantly improve the prognosis of patients with lymph node metastasis, further emphasizing the development of effective theranostics strategies against lymph node metastasis (6–8). Recently, breast cancer has replaced lung cancer as the most common cancer worldwide (9). Similar to other solid tumors, lymph node metastasis plays a major role in promoting the invasion and metastasis of breast cancer. Therefore, reducing the occurrence of lymph node metastasis and removing tumor cells from the lymphatic system are the keys for combating breast cancer.

Current treatment methods for lymph node metastasis mainly include surgical resection, local radiotherapy and chemotherapy. Surgical resection as an invasive treatment have been widely used to perform regional or sentinel lymph node dissection. However, the efficiency of surgical resection usually depends on the diagnostic techniques of lymph nodes. Clinically, methylene blue dye, technetium colloid or fluorescent dye ICG are commonly used to locate and detect sentinel lymph nodes for guiding lymph node resection (10–13). However, these methods have certain limitations. It is difficult to detect the deep lymph nodes and distinguish the lymph node metastasis from normal lymph nodes. According to the literatures, local radiotherapy have been used for advanced patients with metastases to supraclavicular lymph nodes, reducing the risk of local recurrence and improving the overall survival (14, 15). However, the inevitable risks of exposure to radiation of the heart, lungs, and skin can lead to events such as acute radiation skin damage, lung damage, and increased incidence of heart disease (16–19). Similar to radiotherapy, chemotherapy is usually applied in the advanced patients with breast cancer. Recently, the neoadjuvant chemotherapy has been developed to reduce the size of the primary tumor and eliminate minor peripheral lesions before the surgery or radiotherapy, further improving the patient’s prognosis and quality of life (20). Chemotherapeutic agents based on small molecules often result in poor lymphatic absorption in clinical applications, affecting the long-retention in lymph node metastasis (21, 22). More importantly, the systemic side effects of chemotherapy are very obvious (23). Therefore, it is an urgent need to develop new personalized theranostics treatment of lymph node metastasis in breast cancer.

Given its suitable size and surface properties, nanomaterials can freely enter the intercellular matrix (24). During the competitive absorption process between lymphatics and blood vessels, some nanomaterials will preferentially enter the lymphatic vessels from the interstitium (25–27). Therefore, interstitially administration (subcutaneous, intratumoral or peritumoral) of nanodrugs exhibit great potential to target the lymphatic system. The delivery of nanodrugs through the lymphatic system effectively targets and accumulates in lymph node metastasis, avoiding the rapid drug clearance caused by direct ingestion by the blood system and further reducing the risk of toxicity. Utilizing the nanomaterials as nano-drug carriers could optimize drug accumulation in solid tumor sites, achieving highly efficient and selective lymphatic system enrichment by subcutaneous administration (28–30). Although numerous papers have reported that nano-drug combined with small molecular drugs using the principle of lymphatic transport could show the draining lymph nodes and kill the cancer cells aggregated in the lymph node site, theranostic nanoplatform in combination with radioisotope therapy for lymphatic system has been rarely reported (31).

**Scheme 1:**
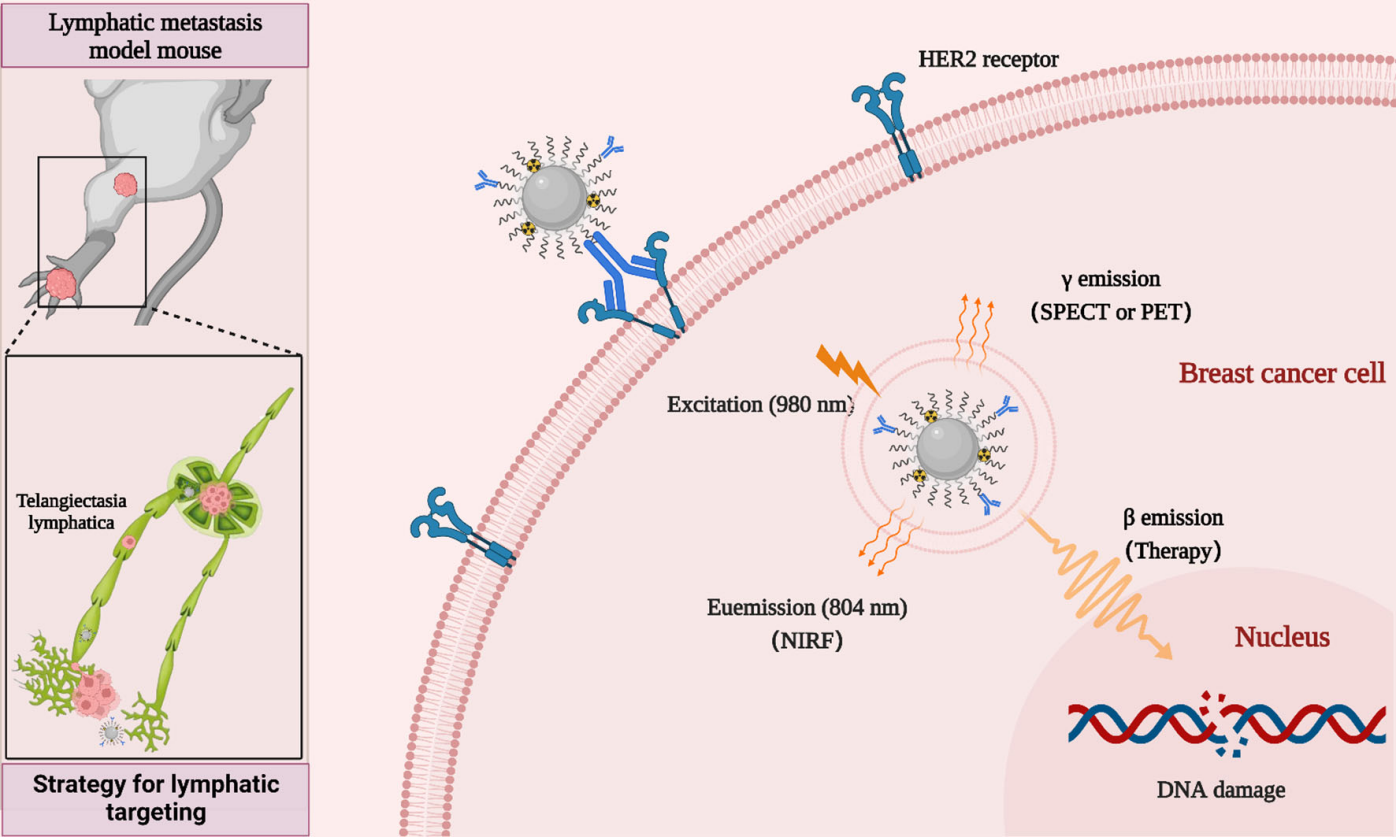
Schematic diagram of strategy for lymphatic targeting and theranostic nanoplatform.

The passive targeting of nano-drugs based on the enhanced penetration and retention (EPR) effect is strongly influenced by tumor heterogeneity. For the metastatic tumor, nano-drugs without tumor targeting ability could induce the non-specific distribution and unnecessary side effects (32, 33). An effective way to overcome these passive targeting limitations is to introduce targeting ligands or antibodies on the surface of nanoparticles for improving the cancer cells uptake of nanodrugs through their active binding ability to receptors or antigens specifically expressed on the tumor cell surface (34–36). Human epidermal growth factor receptor 2 (HER2)-positive breast cancer is an aggressive type of breast cancer that tends to grow more rapidly and spread more easily. Anti-HER2 therapies such as Herceptin (trastuzumab), are highly effective in the clinic, significantly improving the prognosis of patients with HER2-positive breast cancer (37, 38). In addition, trastuzumab has been widely used in the development of various nanodrugs for the diagnosis and treatment of HER2-positive breast cancer (39, 40). Therefore, in this study, we developed rare-earth upconversion nanoprobe (NP-mAb) conjugated with trastuzumab. These NP-mAb could be efficiently labeled with diagnostic radioisotope ^68^Ga (half-life: 68 min) and therapeutic radioisotope ^177^Lu (half-life: 6.71 d) through simple chelation. The obtained nanonuclear drug (^68^Ga-NP-mAb and ^177^Lu-NP-mAb) and nanoprobe can be moved into the lymph node metastasis *via* the delivery of lymphatic system, which developed a new theranostic strategy for lymphatic targeting used for near-infrared fluorescence (NIRF), single-photon emission computed tomography (SPECT), positron emission tomography (PET) and targeted radionuclide therapy (TRT) of HER2-positive breast cancer lymph node metastasis tumor in mice (**Scheme 1**). TRT based on ^177^Lu-NP-mAb could effectively inhibit the occurrence of lymph node metastasis and the growth of tumour in the footpad area and lymph node. Importantly, such therapeutic strategy our developed even exhibited no obvious side effects on the blood system, liver and kidney function of mice. Therefore, our developed strategy will provide a new method for theranostic of lymphatic metastases.

## Results and discussions

**Figure 1:**
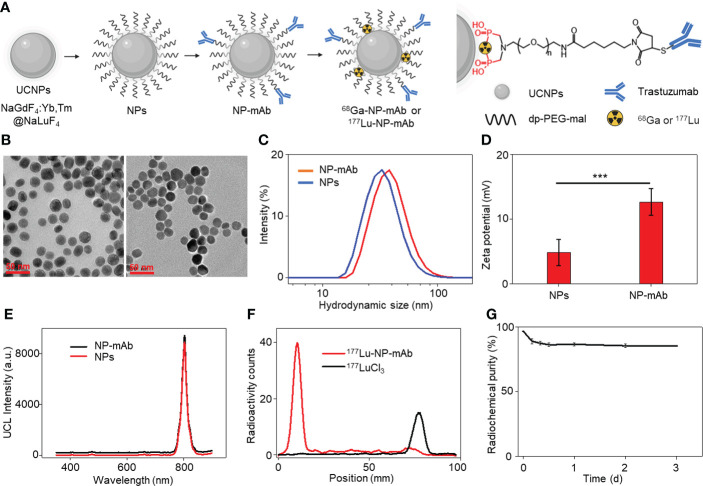
Synthesis and characterization of theranostic nanoplatform. **(A)** Schematic depicting the preparation of theranostic nanoplatform and structure diagram. **(B)** Transmission electron microscopy (TEM) image of NPs and NP-mAb nanoparticles. **(C)** Hydrodynamic size of NPs and NP-mAb in water determined by DLS. **(D)** Zeta potential of NPs and NP-mAb in water (***P< 0.001). **(E)** UV–vis spectra of NPs and NP-mAb under 980 nm light irradiation. **(F)** Radiochemical pure analysis of ^177^Lu-NP-mAb and ^177^LuCl_3_. **(G)** Radiolabeling stability of 177Lu-NP-mAb in PBS solution.

We began the study with the synthesis of rare-earth upconversion nanoparticles (UCNPs, NaGdF_4_:Yb,Tm@NaLuF_4_) *via* a liquid-solid-solution (LSS) solvothermal method (**Figure 1A**) (41–44). Then, UCNPs were chelated with bisphosphate-headed polyethylene glycol (PEG) ended with a maleimide group (dp-PEG-mal) to get water-soluble nanoparticles (i.e., NPs). To obtain the lock-and-key specific targeting ability toward HER2-positive breast cancer lymphatic metastasis, trastuzumab was conjugated with PEGylated NPs to obtain NP-mAb *via* a reaction between sulfhydryl residues of antibody and maleimide groups on the terminal PEG. Finally, positron-emitting ^68^Ga and β-emitting ^177^Lu radionuclides were labeled *via* chelating with bisphosphate groups to yield the final multimodal theranostic nanoparticles ^68^Ga-NP-mAb and ^177^Lu-NP-mAb, respectively. Transmission electron microscopy (TEM) characterization showed the spherical morphology of NPs (**Figure 1B**, left panel) and NP-mAb (**Figure 1B**, right panel) with an average size of 21.99 ± 2.88 nm and 22.5 ± 2.94 nm, respectively (**Figure S1**). Dynamic light scattering (DLS) analyses showed a narrow hydrodynamic size distribution of NPs with an average size of 50 nm, suggesting the hydrophobic UCNPs were successfully converted into hydrophilic UCNPs through the replacement of the oleic acid ligands-functionalized oil-dispersible UCNPs with hydrophilic PEG-coated UCNPs. A slight larger hydrodynamic size of 60 nm relative to PEGylated NPs was observed for NP-mAb (**Figure 1C**). Moreover, the zeta potential of the PEGylated NPs before and after antibody conjugation was changed from 4.81 to 12.63 mV (**Figure 1D**). These results strongly demonstrated that the monoclonal antibody molecules were successfully coupled on the surface of the NPs. Of note, antibody modification didn’t influence the optical spectrum of NPs and NP-mAb with exhibiting a near-infrared emission centered at 804 nm under the excitation of 980-nm laser (**Figure 1E**). In addition, thin-layer paper chromatography assay indicated the radiolabeling yield of both ^68^Ga-NP-mAb and ^177^Lu-NP-mAb reached 95% with ideal radiolabeling stability as well as excellent stability for NP-mAb in PBS and 10% FBS (**Figures 1F, G** and **Figures S2** and **S3**). Besides, the stability of NP-mAb in different solutions were investigated.

**Figure 2:**
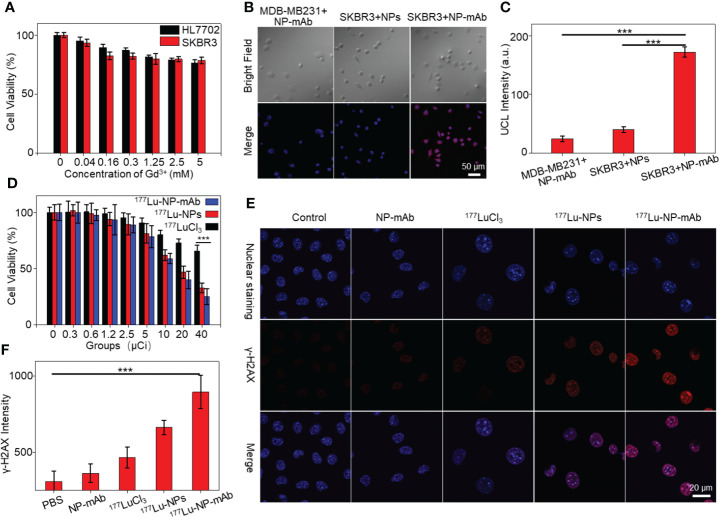
Cell experiments. **(A)** The cytotoxicity of NP-mAb at different concentrations. **(B)** Confocal imaging of the HER2 over-expressed cell line (SKBR3) and HER2 low-expressed cell line (MDA-MB-231) incubated with the NPs and NP-mAb, respectively (blue: Nuclear staining, red: UCL Channel, the embedded scale bars correspond to 50 μm). **(C)** UCL Intensity of confocal imaging in cell binding experiment (***P< 0.001). **(D)** Relative viabilities of SKBR3 cells treated with different doses of free ^177^Lu, ^177^Lu-NPs and ^177^Lu-NP-mAb for 24 h. Asterisks indicate statistical significance (***P< 0.001). **(E)** γ-H2AX fluorescence images (blue: Nuclear staining, red: γ-H2AX) of SKBR3 cells with different treatments. **(F)** γ-H2AX Intensity of confocal imaging in DNA damage (***P< 0.001).

We next studied the cytotoxicity of NP-mAb to HER2-positive human breast carcinoma cell line SKBR3 cells and normal human liver cell line HL7702 cells by the CCK-8 assay. As shown in **Figure 2A**, after incubating with NP-mAb at various concentrations for 24 h, the nanoparticles showed minimized toxicity towards either SKBR3 cells or HL7702 cells, suggesting the suitable biocompatibility of NP-mAb for further *in vivo* study. To investigate the targeting capability of the NP-mAb, the cellular uptake study was conducted. SKBR3 cells with high HER2 expression were incubated with NPs and NP-mAb for 24 h, respectively, and the HER2 negative-expressed triple-negative human breast carcinoma cell line MDA-MB-231 cells were incubated with NP-mAb for 24 h. After incubation, the upconversion luminescence (UCL) signals of cells after different treatments were acquired under 980 nm laser irradiation (**Figure 2B**). The UCL signal of SKBR3 cells treated with NP-mAb presented a distinct signal, which was 4.28-fold and 7.1-fold higher than those of SKBR3 cells treated with NPs and MDA-MB-231 cells treated with NP-mAb, respectively (**Figures 2B, C**). These results proved that NP-mAb had better targeting efficiency towards HER2-positive SKBR3 cells, which was also verified by the UCL images of collected cell pellets after various treatments (**Figure S4**).

To investigate the cytotoxicity effect of ^177^Lu-NP-mAb, SKBR3 cells were incubated with different concentrations of ^177^LuCl_3_, ^177^Lu-NPs, and ^177^Lu-NP-mAb for 24 h, respectively. The cell viabilities were measured by CCK-8 assay. As shown in **Figure 2D**, the killing effect of ^177^Lu-NP-mAb on SKBR3 cells was significantly higher than that of ^177^LuCl_3_ and ^177^Lu-NPs at all detected radioactive dosage attributing to the trastuzumab-mediated higher targeting efficiency of ^177^Lu-NP-mAb relative to control groups. Meanwhile, the DNA damage of SKBR3 cells treated with ^177^Lu-NP-mAb nanoparticles was also studied (**Figure 2E**). The γ-H2AX luminescence imaging showed that ^177^Lu-NP-mAb induced significantly higher DNA damage than other control groups (**Figure 2F**).

**Figure 3:**
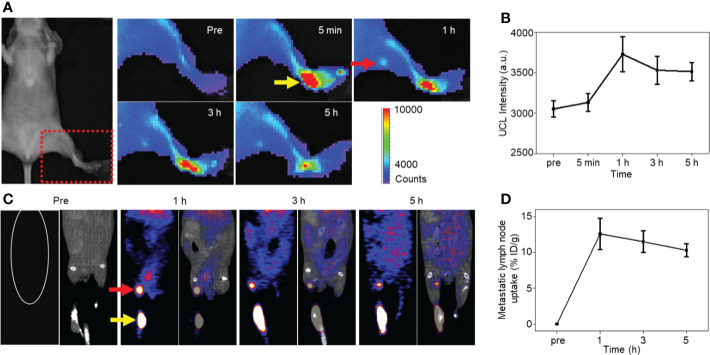
*In vivo* imaging experiments. **(A)** NIRF imaging of mice model bearing SKBR3 were obtained at different time points of NP-mAb injection (the red arrow indicates the site of metastatic lymph node, the yellow arrow indicates the injection site in foot pad). **(B)** Changes of upconversion luminescence signal in lymphatic metastasis after NP-mab injection. **(C)** PET imaging of mice model bearing SKBR3 were obtained at different time points of ^68^Ga-NP-mAb injection (red arrow: lymphatic metastasis, yellow arrow: injection site in foot pad). **(D)** Changes of radioactive ingestion signal in lymphatic metastasis after ^68^Ga-NP-mAb injection.

As follows, NP-mAb and its radioactive counterpart ^68^Ga-NP-mAb were used for *in vivo* imaging of lymphatic metastasis. HER2-positive SKBR3 breast cancer lymphatic metastasis model was established according to the literatures (45, 46). Mice model bearing SKBR3 lymphatic metastasis were intratumorally injected with NP-mAb or ^68^Ga-NP-mAb (5 mCi ^68^Ga/kg) in the foot pad, and imaged by a small animal upconversion luminescence *in vivo* imaging system (IVIS, Lumina XRMS, America) and a small animal positron emission tomography system (micro-PET, Siemens Inveon, Germany), respectively. As shown in **Figure 3A**, an obvious UCL signal was observed in the metastatic lymph node (red arrow) after 1 h injection of NP-mAb in the foot pad (yellow arrow), and the UCL signal was still evident at 5 h post-injection of nanoparticles. Time-dependent UCL signal changes in metastatic lymph node are shown in **Figure 3B**, indicating that NP-mAb can accumulate into the metastatic lymph node efficiently within 1 h post-injection of nanoparticles and enable a long-term longitudinal imaging window. Besides, for radioactive imaging, as displayed in **Figure 3C**, a significant radioactive signal was observed in metastatic lymph node after 1 h injection of ^68^Ga-NP-mAb in foot pad. Quantitative analyses showed similar result with UCL imaging and the highest uptake of the metastatic lymph node reached 12.6 ± 2.2% ID/g at 1 h post-injection of nanoparticles (**Figure 3D**). This further demonstrates that the nanoprobes can be transported through the lymphatic system and specifically gathered in the metastatic lymph node.

**Figure 4:**
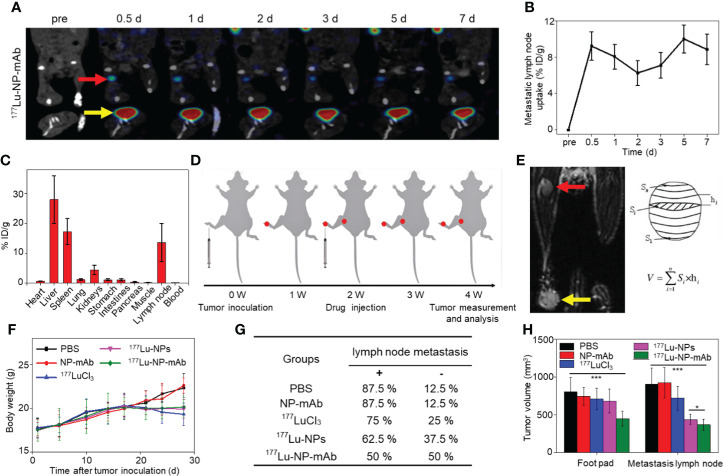
*In vivo* therapeutic efficacy by radiotherapy. **(A)** SPECT imaging of SKBR3 tumor bearing mice with ^177^Lu-NP-mAb injected in the left foot pad at different time points (the red arrow indicates the site of metastatic lymph node, the yellow arrow indicates the injection site in foot pad). **(B)** Uptake curves of ^177^Lu-NP-mAb in lymphatic metastasis at different time points. **(C)** *In vivo* biodistribution of ^177^Lu-NP-mAb in major organs and tissues at 24 h after injected in the foot pad (error lines represent mean ± standard deviation, n = 3). **(D)** Schematic diagram of radioisotope therapy to suppress primary tumor and metastasis lymph nodes growth. **(E)** MRI imaging of lymphatic metastasis model mouse and Schematic diagram of tumor volume calculation method. **(F)** Changes in body weight of mice in each group during the therapeutic cycle. **(G)** Lymph node metastasis rate of different groups of mice (PBS, NP-mAb, ^177^LuCl_3_, ^177^Lu-NPs, and ^177^Lu-NP-mAb, n = 8 for each group) after 2 weeks of radioisotope therapy (**+** indicates positive lymph node metastasis in mice, - indicates negative lymph node metastasis in mice). **(H)** The volume of primary tumor and metastatic lymph nodes in different groups of mice after 2 weeks of radioisotope therapy. Asterisks indicate statistical significance (***P< 0.001, *P< 0.05).

Before determining *in vivo* therapeutic effect of ^177^Lu-NP-mAb, *in vivo* behavior of ^177^Lu-NP-mAb in mice bearing SKBR3 lymphatic metastasis was investigated. Notably, except for β-ray emission for therapy, ^177^Lu emits γ-ray emission (208 keV and 113 keV) with long radioactive half-time (6.7 day), enabling long-term monitoring the *in vivo* behavior of ^177^Lu-NP-mAb in the metastatic lymph node by a small animal single-photon emission computed tomography imaging system (micro-SPECT, MILabs, Netherlands). As shown in **Figure 4A**, ^177^Lu-NP-mAb-treated mice exhibited significant accumulation and prolonged retention of radionuclides in the lymphatic metastatic site (red arrow) with the uptake as high as 8.84 ± 1.68% ID/g even at 7 d post-injection of ^177^Lu-NP-mAb shown in **Figure 4B**. Besides, *in vivo* biodistribution was also investigated at 24 h post injection of ^177^Lu-NP-mAb in the footpad of mouse model (**Figure 4C**). We found that ^177^Lu-NP-mAb exhibited an obvious accumulation (13.5 ± 6.38%ID/g) in lymphatic metastasis at 24 h except for the liver and spleen uptake. These results showed that the nanoprobe had good targeting performance in metastatic lymph nodes. In addition, there was a large amount of radioactive accumulation in the liver and spleen, which was related to the characteristics of nanoparticles and their easy uptake by the monocyte macrophage system (MPS).

Based on the excellent accumulation and retention of ^177^Lu-NP-mAb in the metastatic lymph node, the therapeutic effect of ^177^Lu-NP-mAb on primary and metastatic tumors was evaluated. The timeline of construction of tumor model, therapeutic treatment, and outcome analyses were shown in **Figure 4D**. After 2 weeks of inoculation of the tumor cells into nude mice, mice were injected with PBS, NP-mAb, ^177^LuCl_3_, ^177^Lu-NPs, or ^177^Lu-NP-mAb in the footpad, respectively. After therapies, tumor volumes of both primary (yellow arrow, injection site in the foot pad) and metastatic lymphatic tumors (red arrow) were detected and calculated through small animal magnetic resonance imaging system (MRI, MRS3000, MR Solution, Britain) shown in **Figure 4E**. No obvious weight loss for all treatment groups (**Figure 4F**). According to the MRI data in **Figure S5** and the analyses in **Figure 4G**, among all the treatment groups, the mice treated with ^177^Lu-NP-mAb possessed the lowest risk of lymph node metastasis with metastatic occurrence probability decreased from 87.5% in PBS-treated group into 50% in ^177^Lu-NP-mAb-treated group. Additionally, compared with PBS-treated group, the rate of lymph node metastasis was slightly decreased in the ^177^LuCl_3_- treated group, which might be due to the antitumor effect of β-rays emitted by ^177^Lu. In contrast, it was easier to understand that there was no change in the metastatic rate of lymph nodes in the NP-mAb treated group relative to PBS-treated group. We also collected the primary tumor and lymph node metastasis to record the volume of tumor using MRI (**Figure S5**). As shown in **Figure 4H**, the primary tumor volumes of mice treated with ^177^Lu-NP-mAb was smaller than that of the other four groups, which might be related to the better distribution and intracellular uptake of ^177^Lu-NP-mAb within the tumor. From the lymph node metastasis volume analysis, both ^177^Lu-NPs and ^177^Lu-NP-mAb could inhibit the tumor growth, suggesting that the nanoparticles could enter into the lymphatic system and accumulate in the lymph node. Moreover, the inhibitory effect of ^177^Lu-NP-mAb group was better than that of the ^177^Lu-NPs group (Average volume of lymph node metastasis: 360.76 ± 64.72 mm^3^ to 448.53 ± 43.7 mm^3^, P<0.05), demonstrating that the improved accumulation and retention of nanoparticles in lymph nodes by anti-HER2 antibody could enhance the therapeutic efficacy.

**Figure 5:**
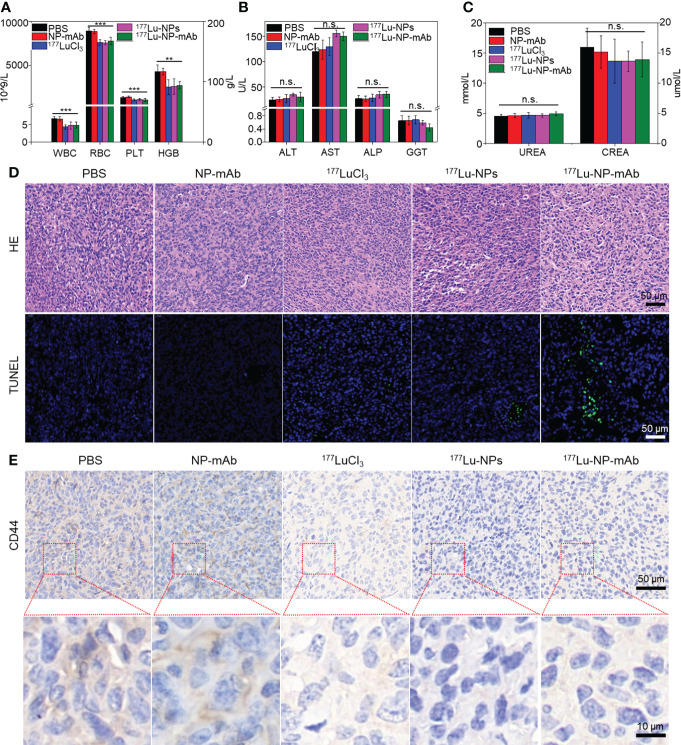
Histological evaluation and safety assay. **(A)** The blood routine tests of mice in different groups after 2 weeks post treatment. Asterisks indicate statistical significance (**P< 0.01, ***P< 0.001). **(B)** The liver function of mice in different groups after 2 weeks post treatment (n.s. indicates no significance). **(C)** The kidney function of mice in different groups after 2 weeks post treatment (n.s. indicates no significance). **(D)** Micrographs of H&E and TUNEL stained tumor slices from metastasis lymph nodes of mice with different groups at 2 weeks after treatment. Scale bar: 50 μm. **(E)** Micrographs of CD44 stained tumor slices from metastasis lymph nodes of mice with different groups at 2 weeks after treatment. Scale bar: 50/10 μm.

The potential side-effect of ^177^Lu-NP-mAb was also investigated. The blood samples collected from mice after different treatments were used to evaluate the routine blood tests, liver and kidney function. As shown in **Figure 5A**, there was a slight decrease in blood indexes such as white blood cell count (WBC), red blood cell (RBC), platelet count (PLT) and hemoglobin (HGB) in ^177^Lu-, ^177^Lu-NPs- and ^177^Lu-NP-mAb-treated groups, which was probably caused by radionuclides-induced bone marrow suppression. Further study showed that WBC, RBC, PLT and HGB have rapidly reduced to a certain extent at the first week after treatment with ^177^Lu-NP-mAb, which kept stable at the next week (**Figure S6**). Such phenomenon are basically consistent with the changes of blood routine level in clinical radionuclide treatment, indicating that radionuclides-mediated bone marrow suppression mainly occurred during the first-week treatment and thus suggesting that prevention should be carried out before treatment to prevent the occurrence of bone marrow suppression. In addition, there are no obvious of liver and kidney toxicity among all the groups using liver function indexes including alanine aminotransferase (ALT), aspartate aminotransferase (AST), alkaline phosphatase (ALP), and gamma glutamyl transpeptidase (GGT) and renal function indexes blood urea (UREA) and creatinine (CREA) (**Figures 5B, C**). Therefore, ^177^Lu-NP-mAb we designed could act as an excellent therapeutic agent for lymph node metastasis with minimal side-effects.

Besides, we also investigated the potential mechanism of radioisotope therapy in lymph node metastasis. Lymph nodes from mice with different treatments were collected for HE staining, TUNEL staining and CD44 immunohistochemistry (IHC) after 2 weeks post injection. HE staining and TUNEL staining of lymph nodes exhibited that^177^Lu-NP-mAb induced most severe apoptosis compared with other control groups (**Figure 5D**). CD44 is widely expressed on the surface of breast tumor stem cells (47). CD44 IHC results of lymph node metastasis showed that CD44 expression level in the ^177^Lu-NP-mAb-treated group was significantly decreased compared with the PBS-treated group, indicating that the tumor stem cells in lymph node metastasis decreased after treatment with ^177^Lu-NP-mAb (**Figure 5E**). The flow cytometry of tumor cells collected from mice after different treatments also indicated the reduced CD44 expression in ^177^Lu-NP-mAb-treated group (**Figure S7A**). In addition, epithelial cell adhesion molecule (EpCAM) expressed on the surface of most epithelial tumor cells, including breast cancer cells, is an important marker in circulating tumor cell detection (CTC). Flow cytometry results showed that the expression level of EpCAM in the ^177^Lu-NP-mAb-treated group was significantly lower than that in PBS-treated group **(** **Figure S7B** **)**. Therefore, ^177^Lu-NP-mAb could significantly inhibit the lymph node metastasis and reduce the incidence of tumor metastasis.

## Conclusion

In this work, we successfully designed a nanoprobe conjugated trastuzumab based on upconversion nanoparticles, further developed a nanonuclear drug labeled ^68^Ga or ^177^Lu, adopted a new imaging and theranostic strategy for lymphatic targeting, to realize the multimodal imaging and theranostics of lymph node metastasis. NIRF/PET/SPECT imaging showed that nanoprobe exhibited high accumulation and prolonged retention in lymph node metastasis. Importantly, the injected nanonuclear drug significantly reduced the occurrence of lymph node metastasis and inhibited the growth of lymph node metastasis. In addition, nanonuclear drug induced no obvious side-effect to treated mice though the blood routine, liver and kidney function assay. Therefore, this study not only provides a versatile nanoplatform for the applications of multimodal imaging and theranostics but also validates new strategy for lymphatic metastasis targeting by delivery of nanodrugs through the lymphatic system, which is meaningful to guide the exploration and advances of more effective theranostics strategies against tumors.

## Materials and methods

### Preparation of ^68^Ga-NP-mAb and ^177^Lu-NP-mAb

The detailed preparation of NaGdF_4_:Yb,Tm@NaLuF_4_ nanoparticles was provided in the Supporting Information. NP-mAb conjugation was prepared according to the previously reported methods (48). The ^68^Ge/^68^Ga generator (ITG, Germany) was eluted with 4 mL of 0.05 M HCl, and take the middle 2 ml of ^68^Ga for labeling. ^68^Ga solution (1 ml, 185-222 MBq) dissolved in 250 µL of 0.25 M sodium acetate (NaOAc) was added into the NP-mAb solution (1 mg/mL, 0.1 mL) and then stirred at room temperature for 30 min. The mixture was centrifuged at 3000 r/min for 5 min three times through 100 K ultrafiltration tube to remove the excess ^68^Ga. The radiochemical purity of ^68^Ga-NP-mAb was measured by paper chromatography (mobile phase: sodium citrate). ^177^LuCl_3_-HCl solution (20 µL, 18.5–37MBq, ITM, Germany) dissolved in 20 µL of 0.25 M sodium acetate (NaOAc) was added into the NP-mAb solution (1 mg/mL, 0.05 mL) and then stirred at room temperature for 30 min. The mixture was centrifuged at 3000 r/min for 5 min three times through 100 K ultrafiltration tube to remove the excess ^177^Lu. The radiochemical purity of ^177^Lu-NP-mAb was measured by paper chromatography (mobile phase: EDTA). All radionuclide related work is carried out under perfect radiological protection.

### *In vitro* experiments

The human breast cancer cell line SKBR3, MDA-MB-231 and human normal liver cell line HL-7702 were obtained from Cell Source Center, Chinese Academy of Science (Shanghai, China). SKBR3 cells were cultured and passaged in Dulbecco’s Modified Eagle Medium (DMEM) supplemented with 10% fetal bovine serum (FBS), penicillin (100 U/mL), and streptomycin (0.1 mg/mL). MDA-MB-231 cells and HL-7702 cells were cultured and passaged in Roswell Park Memorial Institute 1640 Medium (RPMI-1640) supplemented with 10% fetal bovine serum (FBS), penicillin (100 U/mL), and streptomycin (0.1 mg/mL).

For *in vitro* potential toxicity assay, SKBR3 cells and HL-7702 cells were seeded in 96-well plates (5 × 10^3^ cells/well) and cultured at 37°C overnight, respectively. The different concentrations of NP-mAb (0, 0.04, 0.16,0.31,1.25,2.5 and 5 mM) were added and cultured for 24 h. The cell culture medium was replaced by fresh medium in each well, and the cells were incubated for another 24 or 48 h. Cell viability was measured by the Cell Counting Kit-8 (CCK-8, Bimake, cat.no.B34304) assay.

For *in vitro* radioisotope therapy, SKBR3 cells were seeded in 96-well plates (5 × 10^3^ cells/well) and cultured at 37°C overnight. The different concentrations of ^177^LuCl_3_, ^177^Lu-NPs and ^177^Lu-NP-mAb (0, 0.3,0.6, 1.2,2.5,5,10,20 and 40 μCi) were added and then incubated for 24 h. The cell culture medium was replaced by fresh medium in each well, and the cells were incubated for another 24 h. Cell viability was measured by the CCK-8 assay.

For cell uptake experiments, SKBR3 cells and MDA-MB-231 cells were seeded onto a glass-bottom cell culture dish (Φ15 mm, NEST) at densities of 5 × 10^4^ cells/well, respectively. After 12 h, SKBR3 cells were treated with NP-mAband NPs, respectively. MDA-MB-231 cells were treated with NP-mAb. After another 12 h of incubation, cells were stained with 4’,6-diamidino-2-phenylindole (DAPI) and imaged by confocal microscopy (FV1200, OLYMPUS, Tokyo, Japan).

For γ-H2AX (Immunoway, cat.no.YT2154) staining studies, SKBR3 cells were seeded onto a glass-bottom cell culture dish (Φ15 mm, NEST) at densities of 5 × 10^4^ cells/well. Cells were treated with PBS (control), NP-mAb, ^177^LuCl_3_, ^177^Lu-NPs and ^177^Lu-NP-mAb, respectively. After 12 h of incubation, cells were stained with γ-H2AX and DAPI and then imaged by confocal microscopy (FV1200, OLYMPUS, Tokyo, Japan).

### *In vivo* experiments

Female BALB/c nude mice (5-8 weeks) were purchased from Changzhou Cavens Company. All animal experiments were performed according to the experimental animal protocols of Soochow University, and the experiments were approved by the animal ethics committee of Soochow University.

To create the HER2-positive breast cancer lymphatic metastasis model, a single-cell suspension of 2 × 10^6^ SKBR3 cells in 20 μL PBS was injected into the left footpad area of BALB/c nude mice. The popliteal lymph nodes could be palpated for enlargement and hardening after about 2 weeks, demonstrating that SKBR3 cells were successfully metastasized to the popliteal lymph nodes.

For *in vivo* NIRF imaging, mice bearing SKBR3 tumor were injected in the footpad with NP-mAb (120 μg/20 μl). NIRF imaging were acquired by IVIS at different time points before and after injection (Pre, 5 min, 1h, 3h, 5h). The imaging data were analyzed using Living ImageⓇ4.5 (PerkinElmer). For *in vivo* PET imaging, mice bearing SKBR3 tumor were injected into the footpad with ^68^Ga-NP-mAb at radioactive dose of 100 μCi. Micro-PET was performed at different time points after injection (1h, 3h, 5h; n = 3). The imaging data were reconstructed and analyzed using Inveon Workplace (Siemens). The imaging data were reconstructed by 3-dimensional ordered subsets expectation maximum (3D OSEM) algorithm using Inveon Workplace (Siemens) without correction for attenuation or scatter. The CT data from the PET/CT examination were reconstructed in the coronal plane as 0.1-mm-thick sections. The following parameters were used for imaging: 80 kV, 100 mAs, 0.32 s per rotation. The imaging-derived percentage injected dose per gram (%ID/g) of lymphatic metastasis were calculated at Inveon Research WorkStation.

For *in vivo* SPECT imaging and bio-distribution studies, mice bearing SKBR3 tumor were injected into the footpad with ^177^Lu- NP-mAb or ^177^Lu- NPs at radioactive dose of 50 μCi. SPECT was performed using a general-purpose mouse collimator with 2.0 mm pinholes, and >1500 cps/MBq sensitivity with an U-SPECT+/CT imaging system (MILABS) at different time points after injection (Pre, 0.5d, 1d, 2d, 3d, 5d, and 7d; n = 3). Two 10-minute sets of data (frames) were acquired and combined. Pixel-based ordered subset expectation maximization (POSEM) reconstruction was used with 4 subsets, 6 iterations, and a 3D-Gaussian kernel (FWHM of 0.8 mm) filter. X-ray microCT was used for anatomic guidance and attenuation correction. The imaging data were reconstructed using the two-dimensional ordered subsets-expectation maximization algorithm. Volume rendered images were generated using professional PMOD software (MILabs).

Mice bearing SKBR3 lymphatic metastasis model were raised two weeks and then assigned into five groups including 1) PBS (control group), 2) NP-mAb treated group, 3) 177LuCl3 treated group (3.7 MBq per mouse), 4) ^177^Lu- NPs treated group (3.7 MBq per mouse) and 5) ^177^Lu- NP-mAb treated group (3.7 MBq per mouse corresponding to 50 ug of NP-mAb). These samples were injected in the left footpad area (n = 8), while the control group were administrated with 0.02 mL of PBS (n = 8). After another two weeks, micro-MRI was used to measure the tumor volume in the foot pads and lymph node metastasis. The tumor volumes were calculated using the formula: $\left(V=\sum_{i=1}^{n}S_{i}\times h_{i}\right)$ (49, 50). When the tumor volume reached 2 cm^3^ or the weight loss exceeded 20%, the experiment was terminated and the animals were sacrificed.

### Safety assessment

For immunofluorescence staining, the lymph node metastasis obtained from mice with different treatments including PBS, NP-mAb, 177LuCl3, ^177^Lu- NPs and ^177^Lu- NP-mAb were sliced and stained with anti-CD44 (abcam, Clone: EPR18668). Hematoxylin and eosin (H&E) staining and terminal deoxynucleotidyl transferase dUTP nick end labelling (TUNEL, Roche, cat.no.11684817910) assay also were carried out to evaluate morphologic changes and apoptosis of SKBR3 tumor cells. The mice were killed by cervical dislocation and the lymphatic metastases were surgically removed immediately. Then, the tissue samples of lymphatic metastases were fixed in 10% formalin before being stained and analyzed for pathological changes.

The blood routine, liver function and kidney function were investigated to evaluate the side effects of mice with different treatment. Blood routine assay including WBC, RBC, PLT and HGB were measured by XN-1000 F Automated Hematology Analyzer (KOBE, JAPAN). Liver and kidney functions including ALT, AST, ALP, GGT, UREA and CREA were measured by Clinical Chemistry Analyzer BS-420(Mindray, China).

To study the antigen expression of SKBR3 cancer cells *in vivo*, immunocytochemistry was used to detect cell surface antigens. The lymph node metastasis obtained from mice with different treatments including PBS, NP-mAb, 177LuCl3, ^177^Lu- NPs and ^177^Lu- NP-mAb were homogenized in PBS (pH 7.4) containing 1% Fetal Bovine Serum (FBS) to acquire cell suspension. Afterwards, the SKBR3 cells were stained by anti-EpCAM (abcam, Clone: EPR20532-225) or anti-CD44 antibodies for flow cytometry assay.

### Statistical analysis

All results are expressed as means ± SEM or SD as indicated. Significance between multiple groups was determined by one-way ANOVA analysis, t-test was used to compare the two groups of data. All statistical analyses were carried out using SPSS statistical software version 19.0 (IBM Corp., Armonk, NY, USA). P<0.05 was considered to indicate statistically significant differences.

## Data availability statement

The original contributions presented in the study are included in the article/**Supplementary Material**. Further inquiries can be directed to the corresponding authors.

## Ethics statement

The animal study was reviewed and approved by The animal ethics committee of Soochow University.

## Author contributions

The manuscript was written through contributions of all authors. CZ, YZ and ML contributed equally. All authors contributed to the article and approved the submitted version.

## Funding

This work was supported by the National Key Research Program of China (2018YFA0208800), National Natural Science Foundation of China (22076132, 21976128), the Natural Science Foundation of Jiangsu Province (BK20200100, BK20190830), Suzhou Administration of Science and Technology (SYS2020082), Key Talents Program for medical Application of nuclear Technology (XKTJ-HRC20210012) and the Project of State Key Laboratory of Radiation Medicine and Protection, Soochow University (GZK1201806).

## Acknowledgments

We also would like to thank the members in the Zhu Lab at Soochow University and the members in the Wang Lab in Nanjing First Hospital, Nanjing Medical University. Thanks to the Jiangsu Province International Joint Laboratory For Regeneration Medicine.

## Conflict of interest

The authors declare that the research was conducted in the absence of any commercial or financial relationships that could be construed as a potential conflict of interest.

## Publisher’s note

All claims expressed in this article are solely those of the authors and do not necessarily represent those of their affiliated organizations, or those of the publisher, the editors and the reviewers. Any product that may be valuated in this article, or claim that may be made by its manufacturer, is not guaranteed or endorsed by the publisher.
