# Supplementary material for: Near-infrared upconversion multimodal nanoparticles for targeted radionuclide therapy of breast cancer lymphatic metastases
Source: Front Immunol. 2022 Dec 1;13:1063678. doi: 10.3389/fimmu.2022.1063678 (PMC9751193; doi:10.3389/fimmu.2022.1063678)
Supplement: Supplementary file 1 [file DataSheet_1.docx]

**Supporting Information**

**Near-infrared Upconversion Multimodal Nanoparticles for Targeted Radionuclide Therapy of Breast Cancer Lymphatic Metastases**

**Preparation of NaLnF4 (Ln=Gd, Er, Yb) nanocluster precursors:** Firstly, 0.5 mmol LnCl_3_ (Gd, Yb, Tm = 80:18:2) was dissolved in 1 ml of water, and 2 mmol NaF was dissolved in 4 ml of water. 1.2 g of NaOH was weighed and transferred into a 50 mL single-neck flask, then 4 mL of water was added to completely dissolve NaOH. Afterwards, 9 mL of anhydrous ethanol and 20 mL of oleic acid were added into the above-mentioned solution. The mixture was stirred for about 10 min. LnCl_3_ and NaF solution were added into the flask dropwise, and stirred for 1 h. The product was precipitated by adding 2 times the volume of ethanol and centrifuged at 10000 r.p.m. for 10 min, then rinsed with ethanol and cyclohexane. The final product was dispersed in 2 mL of cyclohexane. The same procedure was applied to the preparation of NaLuF4 nanocluster precursors.

**Preparation of NaGdF4:Yb,Tm@NaLuF4 nanocrystals:** 10 mL of octadecene and 6 mL of oleic acid were added into a 100 mL three-neck flask. The prepared NaLnF4 nanocluster precursors dispersed in cyclohexane solution was also added into the three-neck flask. The cyclohexane was removed from the system by stirring at 70 ^o^C for 30 min and bubbling nitrogen. The temperature was increased to 280 ^o^C at a rate of ~10 ^o^C /min. During the heating process, a condenser was added at ~200 ^o^C. The reaction was cooled down to room temperature after 1 h. With the same process above, NaLuF4 nanoparticle precursor was added, and the reaction stood for 1 h before cooling down to room temperature. The precipitate was collected by centrifugation at 10,000 rpm for 5 min and washed once with ethanol, and the product was finally dispersed in cyclohexane.

**PEG surface modification of NaGdF_4_:Yb,Tm@NaLuF_4_ nanoparticles:** To render the hydrophobic nanoparticles biocompatible, asymmetric polyethylene glycol (PEG) bearing a maleimide group at one end and a diphosphate group at the other end, denoted as mal-PEG-dp (Suzhou Xinying Biomedical Technology Co., Ltd), was used to replace the oleate ligand since the diphosphate group possesses a much higher binding affinity to Lu^3+^. 10 mg of hydrophobic UCNP and 3 times the volume of acetone were mixed and centrifuged to precipitate, then the supernatant was discarded, and the precipitate was dissolved in tetrahydrofuran. 100 mg of PEG dissolved in tetrahydrofuran was mixed with UCNP, and the mixture were stirred and reacted at 40 ^o^C for 24 h. After the reaction was completed and cooled down to room temperature, 3 times the volume of cyclohexane was used to precipitate. The nanoparticles were washed twice with cyclohexane. The precipitate was dried in a vacuum oven at room temperature for 4 h. The dried nanoparticles were dissolved in pure water and ultrafiltered through a 100 kDa ultrafiltration tube for three times to remove the excess PEG. The modified UCNP was stored at 4 °C for next experiments.


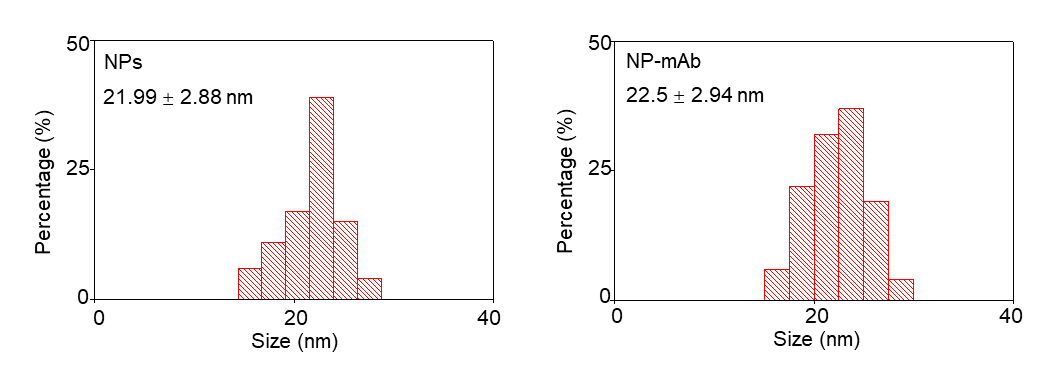


Figure S1: The size distribution of NPs and NP-mAb.


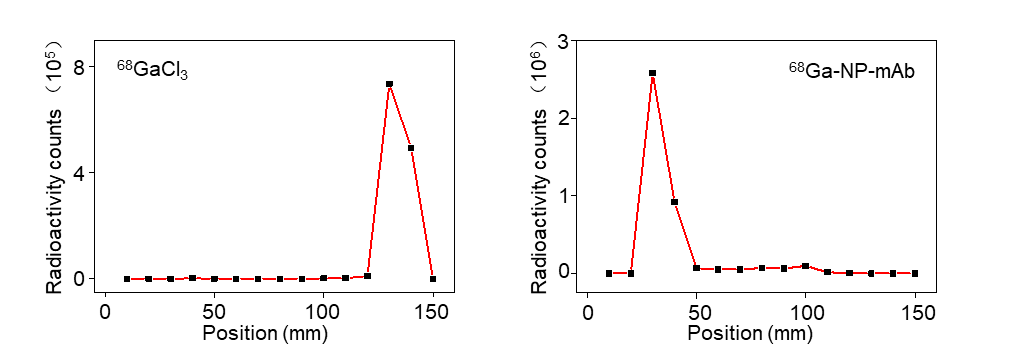


Figure S2: Radiochemical pure analysis of ^68^GaCl_3_ and ^68^Ga-NP-mAb.


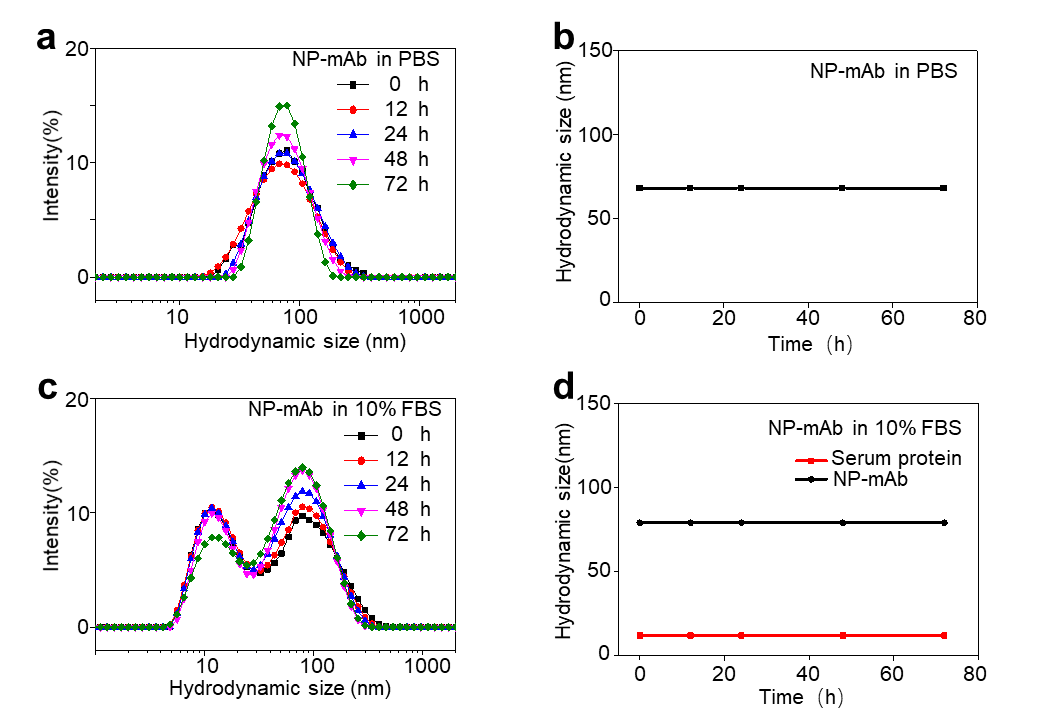


Figure S3: Hydrodynamic size of NP-mAb in PBS (a-b) and 10% FBS (c-d) at different time points determined by DLS. NP-mAb showed a scattering peak in PBS and double scattering peaks in 10% FBS, where the smaller one was the scattering peak of serum protein and the larger one was the scattering peak of nanoparticles.


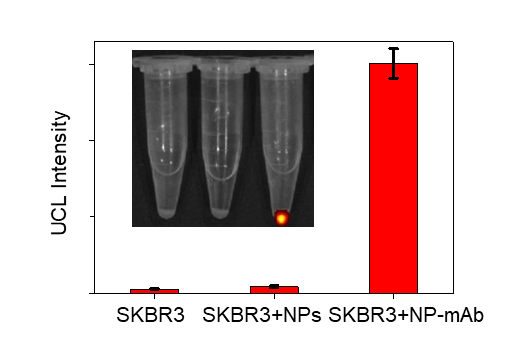


Figure S4. UCL Intensity of IVIS imaging in cell binding experiment.


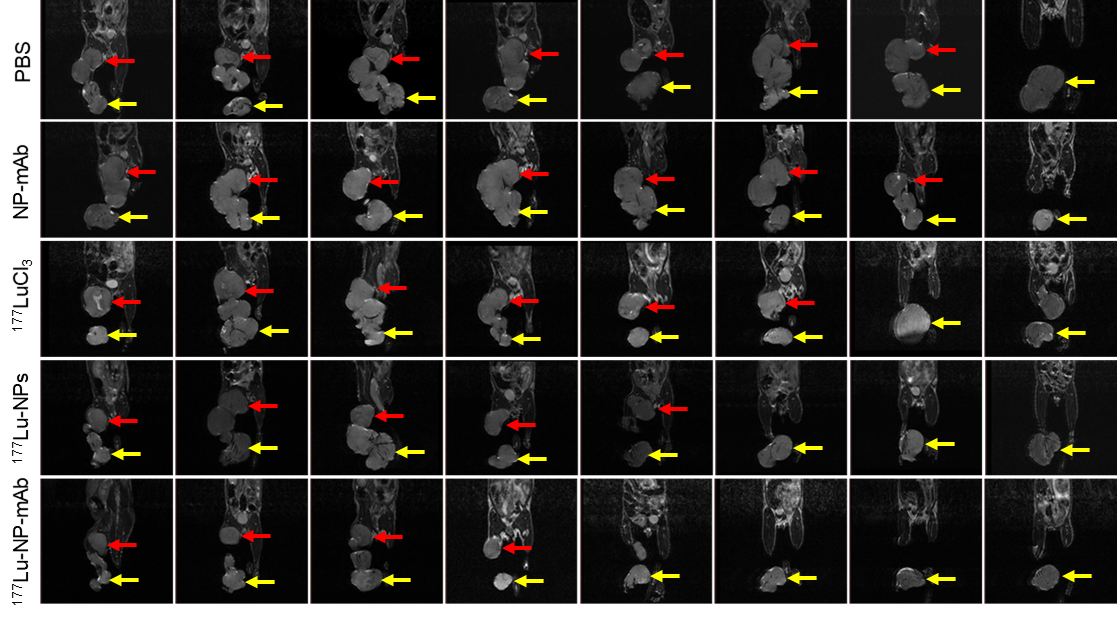


Figure S5. MRI of mice model in different groups were obtained at 2 weeks after treatment (the red arrow indicates the metastatic lymph node, the yellow arrow indicates the primary tumor in foot pad).


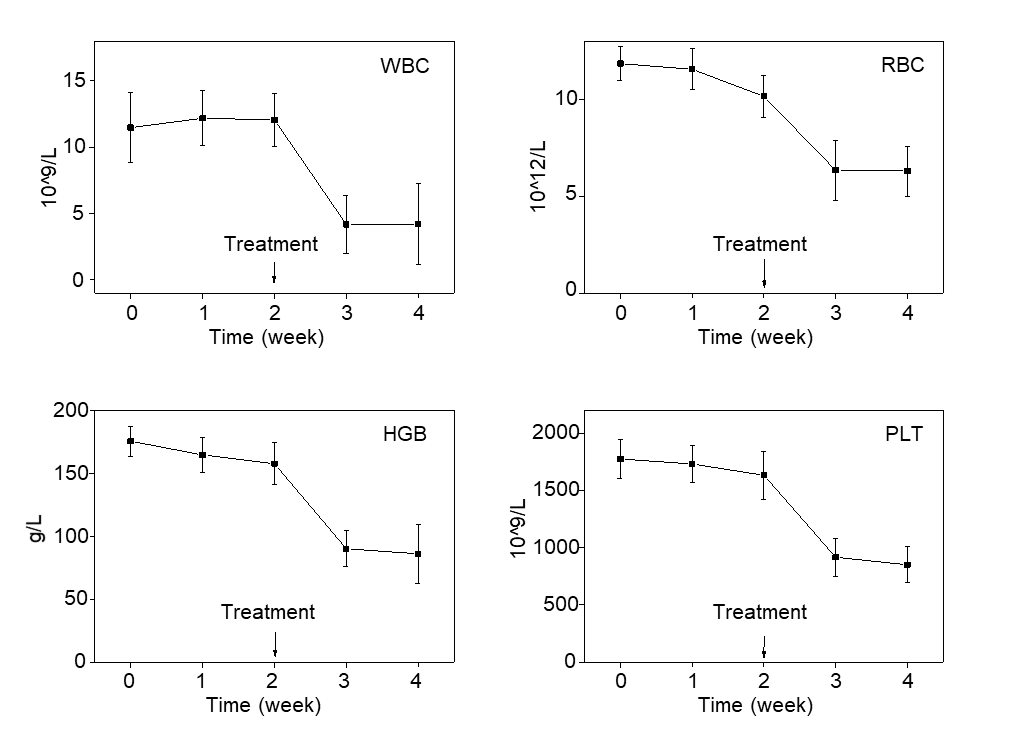


Figure S6. Changes in blood routine tests of mice in ^177^Lu-NP-mAb group during the therapeutic cycle (error lines represent mean ± standard deviation).


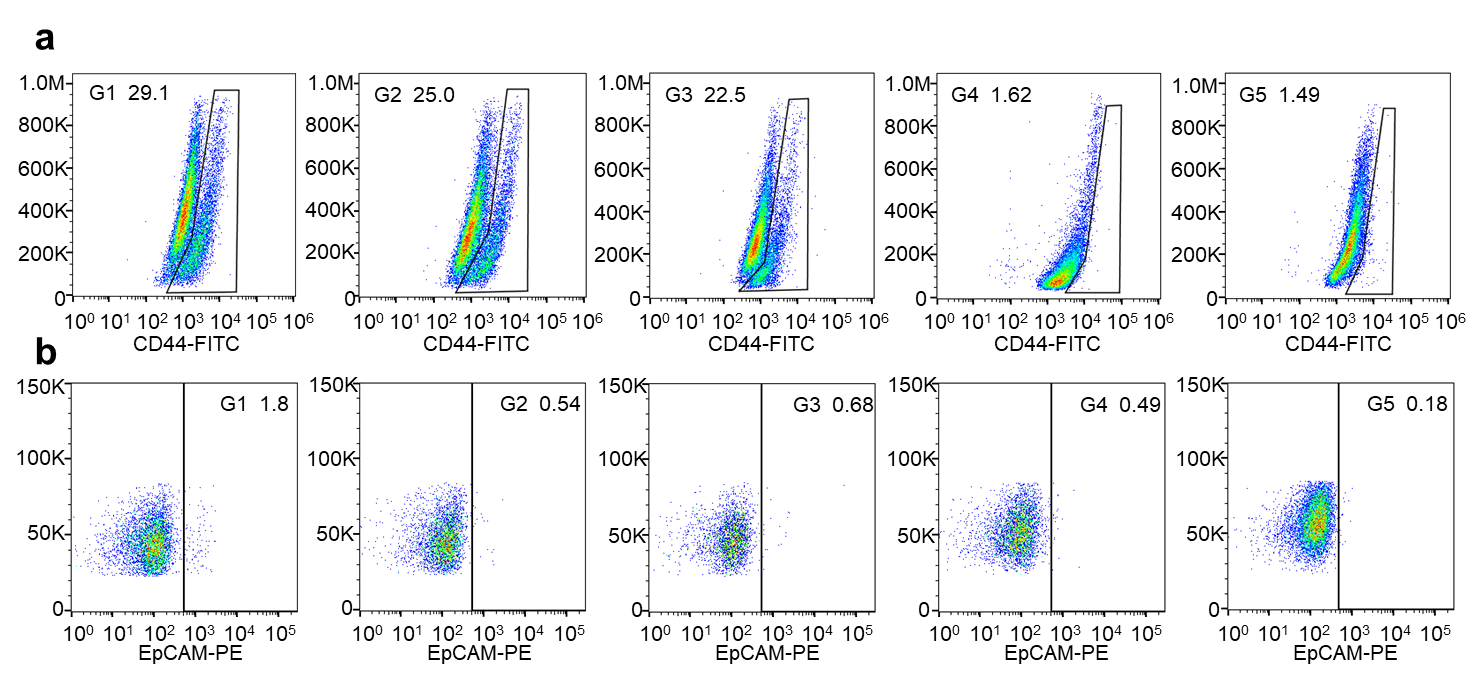


Figure S7. Representative flow cytometry plots showing the CD44 (a) in the metastasis lymph nodes and the EpCAM (b) in the blood from different groups 2 weeks post treatment (G1: PBS, G2: NP-mAb, G3: ^177^LuCl_3_, G4: ^177^Lu-NPs, G5: ^177^Lu-NP-mAb).
